# Differentiation of Benign and Malignant Neck Neoplastic Lesions Using Diffusion-Weighted Magnetic Resonance Imaging

**DOI:** 10.3390/jimaging10100257

**Published:** 2024-10-18

**Authors:** Omneya Gamaleldin, Giannicola Iannella, Luca Cavalcanti, Salaheldin Desouky, Sherif Shama, Amel Gamaleldin, Yasmine Elwany, Giuseppe Magliulo, Antonio Greco, Annalisa Pace, Armando De Virgilio, Antonino Maniaci, Salvatore Lavalle, Daniela Messineo, Ahmed Bahgat

**Affiliations:** 1 Department of Radiodiagnosis, Faculty of Medicine, University of Alexandria, Alexandria 21111, Egypt; omneya.gamaleldin@gmail.com (O.G.); salaheldindesouky@gmail.com (S.D.); dr.sh.shama@gmail.com (S.S.); amelgamaleldin@gmail.com (A.G.); 2 Department of Sensory Organs, “Sapienza” University of Rome, 00184 Roma, Italy; luca.cavalcanti@uniroma1.it (L.C.); giuseppe.magliulo@uniroma1.it (G.M.); antonio.greco@uniroma1.it (A.G.); annalisa.pace@uniroma1.it (A.P.); armando.devirgilio@uniroma1.it (A.D.V.); 3 Department of Medical Oncology and Nuclear Medicine, Medical Research Institute, Alexandria University, Alexandria 21111, Egypt; yasmineelwany@gmail.com; 4 Department of Otolaryngology, Kore University, 94100 Enna, Italy; antonino.maniaci@unikore.it (A.M.); salvatore.lavalle@unikore.it (S.L.); 5 Department of Radiological Sciences, Oncology and Anatomo-Pathological Science, “Sapienza” University of Rome, 00184 Rome, Italy; daniela.messineo@uniroma1.it; 6 Department of Otorhinolaryngology, Alexandria University, Alexandria 21111, Egypt; ahmedbahgat@gmail.com

**Keywords:** squamous cell carcinoma, non-Hodgkin lymphoma, diffusion-weighted MRI

## Abstract

The most difficult diagnostic challenge in neck imaging is the differentiation between benign and malignant neoplasms. The purpose of this work was to study the role of the ADC (apparent diffusion coefficient) value in discriminating benign from malignant neck neoplastic lesions. The study was conducted on 53 patients with different neck pathologies (35 malignant and 18 benign/inflammatory). In all of the subjects, conventional MRI (magnetic resonance imaging) sequences were performed apart from DWI (diffusion-weighted imaging). The mean ADC values in the benign and malignant groups were compared using the Mann–Whitney test. The ADCs of malignant lesions (mean 0.86 ± 0.28) were significantly lower than the benign lesions (mean 1.43 ± 0.57), and the mean ADC values of the inflammatory lesions (1.19 ± 0.75) were significantly lower than those of the benign lesions. The cutoff value of 1.1 mm^2^/s effectively differentiated benign and malignant lesions with a 97.14% sensitivity, a 77.78% specificity, and an 86.2% accuracy. There were also statistically significant differences between the ADC values of different malignant tumors of the neck (*p*, 0.001). NHL (0.59 ± 0.09) revealed significantly lower ADC values than SCC (0.93 ± 0.15). An ADC cutoff point of 0.7 mm^2^/s was the best for differentiating NHL (non-Hodgkin lymphoma) from SCC (squamous cell carcinoma); it provided a diagnostic ability of 100.0% sensitivity and 89.47% specificity. ADC mapping may be an effective MRI tool for the differentiation of benign and inflammatory lesions from malignant tumors in the neck.

## 1. Introduction

The most significant diagnostic challenge in neck imaging is differentiating between benign lesions and malignant malignancies [1]. The ability to differentially diagnose these lesions holds paramount importance, as it empowers clinicians to adopt tailored management approaches for malignant neoplasms [2].

Currently, CT and traditional MRI modalities are widely employed for assessing palpable and non-palpable neck lesions, in addition to supporting their biological characteristics as determined by imaging measures, including necrosis, the invasion of nearby structures, and perineural dissemination [3]. However, the diagnostic efficacy of these modalities is hindered by their reliance on volumetric and morphological criteria, resulting in diminished sensitivity and accuracy, which remain low during diagnostic assessment [2,4,5].

Hence, it is common to see lesions exhibiting inconclusive findings on cross-sectional imaging, thereby prompting the need for additional investigations [3,6].

In the preliminary diagnostic profiling of head and neck cancers, advanced magnetic resonance (MR) techniques, such as proton and phosphorous magnetic resonance spectroscopy (MRS) [7], DCE MRI, and DWI [3,8,9], offer insights into the metabolic, molecular, and pathophysiological features of tumors [2].

Diffusion-weighted imaging (DWI) has been employed for diagnosing and assessing the diagnostic complexities associated with neck-related diseases [1,10,11].

The basic idea behind diffusion-weighted imaging (DWI) is to use the Brownian motion-induced translational motion of water protons in biological tissues. The translational motion causes excited water protons to phase-disperse, which causes signal loss on DWI. This signal loss can be evaluated using the apparent diffusion coefficient (ADC) calculation. The ADC represents the specific biological tissue diffusion capacity [12], which exhibits low values in hypercellular tissues, characteristic of malignant tumors, and elevated ADC values in low-cellularity tissues displayed in non-tumoral tissue alterations, such as edema, inflammation, fibrosis, and necrosis [10].

Our research sought to determine whether adding DWI sequences to traditional MRI improved the ability to distinguish benign and malignant lesions.

## 2. Materials and Methods

MRI studies were conducted for 53 patients presenting with neck lesions to our institution in the period from January 2017 to January 2018. The MR diagnosis was compared to histological results obtained from biopsies or, in some cases, to clinical follow-up information (in four patients).

The study was approved by the Sapienza University Hospital ethical committee (Rif. 6268—Prot. 0306/2021); informed consent was signed by each patient, and all of the study procedures were in accordance with the Helsinki Declaration of 1975, as revised in 1983, for human experimentation. A 1.5 Tesla system (Acheiva 1.5 Tesla, Philips Medical Systems, Amsterdam Nederland) with a 16-channel sensing neurovascular head and neck coil was used for the MR imaging. Furthermore, every participant had a DWI MRI evaluation in addition to standard MRI scans.

The inclusion criteria included patients with clinically detected neck lesions with no specific age group. There were no specific inclusion criteria for the detected lesions, whether they were midline or off-midline, unilateral or bilateral, painful or painless, or multiple or solitary. Exclusion criteria included cystic non-neoplastic lesions, patients with renal impairment, extreme claustrophobia, and patients with metallic foreign bodies.

### 2.1. Conventional MRI

Diffusion-weighted imaging (DWI) was performed after the conventional T1- and T2-weighted imaging procedures.

Before the delivery of contrast material, T1- and T2-weighted images, as well as fat-suppressed (short tau inversion recovery, or STIR) T2-weighted imaging, were obtained.

Contrast-enhanced T1-weighted images (TR/TE of 884/6.8 ms) with fat suppression were acquired after an intravenous bolus injection of gadopentetate dimeglumine (Magnevist^®®^; Bayer Schering Pharma AG, Berlin, Germany) at a dosage of 0.1 mmol per kilogram. The particular area being examined determined how the MRI settings should be set.

### 2.2. DWI MRI

DWI was carried out using a single-shot spin-echo echo-planar imaging (EPI) sequence with the following settings: b-values of 0 and 1000 s/mm^2^, and a 4-mm section thickness for the axial plane, with a field of view (FOV) of 24.0 cm.

The ADC maps were created after the DWIs, and the ROIs identified on the DWIs were transferred onto the matching ADC maps. After that, the ADC values were calculated using specialized workstation software. The ROIs were placed on the lesion’s solid portions, keeping necrotic sections out of the image. The ADC values were obtained from the lesions across different slices.

Three ADC values were found, and statistical analysis was performed using the mean ADC. The Mann–Whitney test was used to statistically compare the mean ADC values between the benign and malignant lesions.

Qualitative assessment of the signal intensity in B1000 images involved the subjective categorization of the lesions as either hypointense (indicative of no restriction) or hyperintense (indicative of restriction). Finding the mean ADC value was a necessary step in the quantitative analysis of the ADC map.

Quantitative ADC map analysis was conducted independently by two radiologists, who exhibited unanimous agreement with no inter-observer discrepancies. During the analysis, the radiologists were blinded to the clinical data and histological results.

#### 2.2.1. Image Analysis

All of the examinations were interpreted clinically at the time of patient presentation by two experienced head and neck imaging radiologists (G.O. and S.S.), with 12 and 22 years of experience in MR head and neck imaging, without knowledge of any other radiologic investigations; final decisions were reached by consensus. The MR imaging findings were then compared with the histopathology or clinical presentations that were performed prior to MRI. The obtained images were reviewed in stack mode on a picture archiving and communications system, and the patterns of diffusion restriction and the ADC values were analyzed.

#### 2.2.2. Statistical Analysis

Data were fed to the computer and analyzed using IBM SPSS software package version 20.0. (IBM Corp., Armonk, NY, USA). Qualitative data were described using number and percent. The Kolmogorov–Smirnov test was used to verify the normality of the distribution. Quantitative data were described using the range (minimum and maximum), mean, standard deviation, and median. The significance of the obtained results was judged at the 5% level. The chi-square test was used for categorical variables for comparison between different groups. Fisher’s Exact or Monte Carlo correction was used for the chi-square test when more than 20% of the cells had an expected count of less than 5. The Kruskal–Wallis test was used for abnormally distributed quantitative variables to compare between more than two studied groups. Receiver operating characteristic curve analysis was used to determine the cutoff point with highest accuracy and the sensitivity as the optimal ADC threshold value to differentiate between benign and malignant lesions, and between non-Hodgkin lymphoma and squamous cell carcinoma. The area under the receiver operating characteristic curve was also calculated. A probability *p*-value of 0.05 indicated a statistically significant difference. Also, the sensitivity, specificity, positive predictive values, and negative predictive values were calculated.

## 3. Results

A total of 53 individuals who presented to our facility with clinically suspected neck lesions were included in this study. Of these, 37 were male (69.8%) and 16 were female (30.2%), with ages spanning from 10 to 85 years (mean age: 47.70 ± 17.85 years). The primary complaints reported by the patients are outlined in Table 1, while the definitive histopathological diagnoses are delineated in Table 2.

The characterization of the lesion in conventional MRI relied on morphological criteria, encompassing signal intensity (SI) variations across different sequences, enhancement following Gd-DTPA administration, and the delineation of lesion margins.

### Overall ADCs

Table 3 displays the mean ADC values of the malignant and benign histological diagnoses. The mean ADC values of the malignant and benign lesions were 0.86 ± 0.28 and 1.43 ± 0.57, respectively. A statistically significant difference was found among the two groups (*p*, 0.001). The mean ADC values of the malignant lesions were significantly lower (*p*, 0.001) than those of the benign lesions.

The average apparent diffusion coefficient (ADC) of the malignant lesions was significantly lower (mean 0.86 ± 0.28) compared to the benign and inflammatory lesions (mean 1.43 ± 0.57).

**Figure 1:**
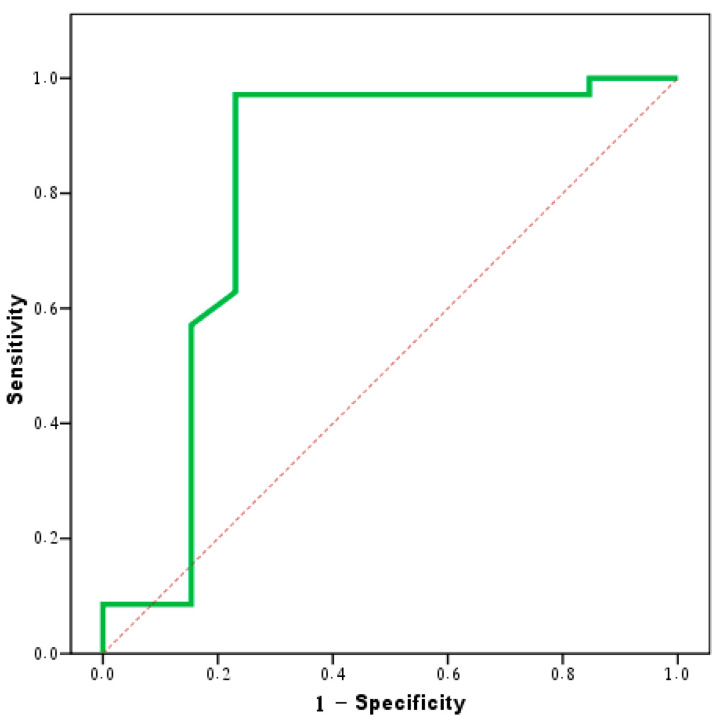
Agreement (sensitivity and specificity) for the average ADC value to predict malignant cases.

The threshold of 1.1 mm^2^/s successfully distinguished between the benign and malignant lesions, with a sensitivity of 97.14%, a specificity of 77.78%, and an accuracy of 86.2% (Table 4 and Figure 1).

Furthermore, a statistically significant distinction was noted in the ADC values of several malignant neck tumors (*p* < 0.001).

**Figure 2:**
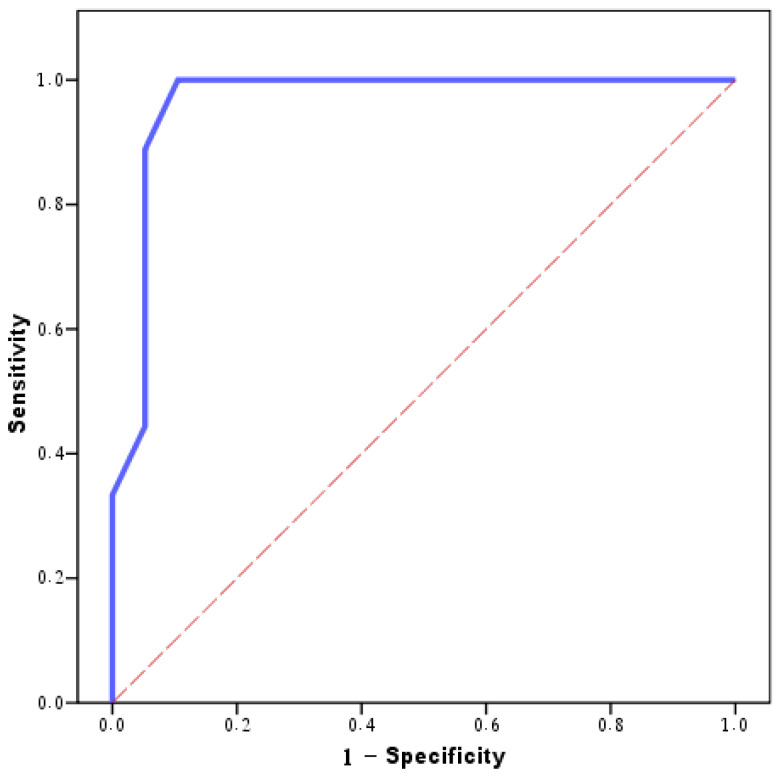
Agreement (sensitivity and specificity) for the average ADC value to predict NHL cases (vs. squamous cell carcinoma).

The NHL group exhibited considerably lower ADC values (0.59 ± 0.09) compared to the SCC group (0.93 ± 0.15). A cutoff value of 0.7 mm^2^/s for the ADC was shown to be the most effective in distinguishing between NHL and SCC. This cutoff point achieved a diagnostic capacity with a 100.0% sensitivity and an 89.47% specificity (Table 5 and Figure 2).

This study included nine patients with suspected benign lesions based on morphological criteria in conventional MRI; they showed a hypointense pattern on DWIs and a correspondingly high ADC value in the ADC map, reflecting their benign nature; four of them were proven by FNAC to be inflammatory tongue ulcers; four of them were proven by excisional biopsy to be schwannoma; one of them was proven to be lymphoid hyperplasia by excisional biopsy. Also, our study included one patient with a suspected malignant lesion based on morphological criteria in conventional MRI; it showed a hyperintense pattern on DWIs and a correspondingly low ADC value in the ADC map, reflecting its malignant nature, which was proven by FNAC to be acinic cell carcinoma of the left sublingual gland. The differentiation of these lesions would have been quite impossible using conventional MRI alone.

This study also included a case suspected to be a parotid malignant lesion based on morphological criteria in conventional MRI; it showed a hyperintense pattern on DWIs and a value of 0.7 in the ADC map, which guided us to the diagnosis of a Warthin tumor rather than a malignant lesion, as a Warthin tumor is characterized by a much lower ADC value than in the case of malignant lesions, and it was proven histopathologically to be a Warthin tumor.

One false-negative case occurred, in which the ADC was 1.8. The patient was complaining of a left neck swelling, clinically suspected to be multinodular goiter; however, his biopsy proved to be medullary carcinoma of the thyroid. One false-positive case was encountered, in which the ADC value was below the cutoff value; however, the final diagnosis proved to be lymphoid hyperplasia.

**Figure 3:**
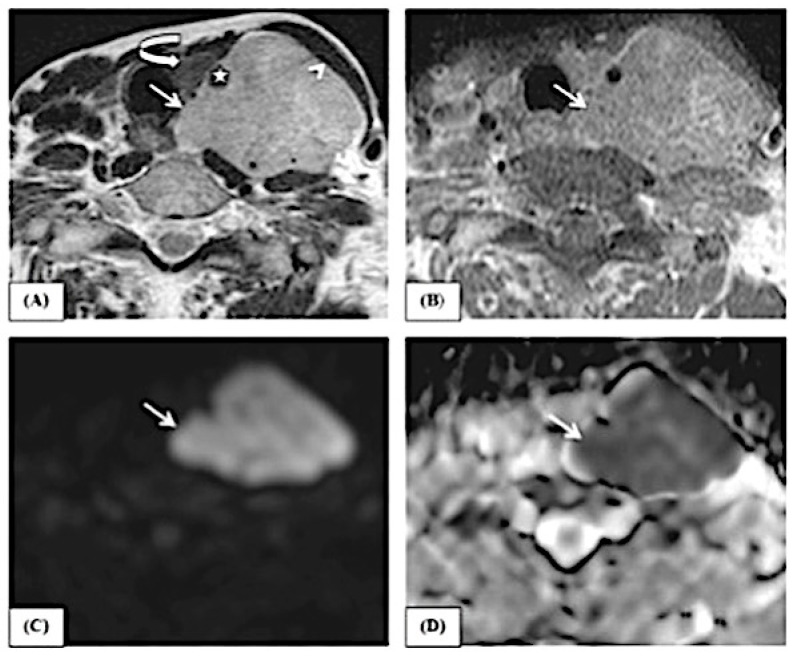
A female patient, 74 years old, complained of a left neck mass. The axial T2 image (**A**) shows a heterogeneous signal of the lesion (white arrow) with nodular infiltrative margins. The lesion is displacing the sternomastoid muscle (arrow head) anteriorly and the thyroid gland (curved arrow) to the right side. It is seen encasing the left common carotid artery (star) and infiltrating the trachea–esophageal groove. The axial T1 post-contrast image (**B**) shows heterogeneous enhancement of the lesion (white arrow). The lesion shows restricted diffusion in the form of a hyperintense signal in B1000 (**C**) (white arrow) and a low ADC value (0.6) in the ADC map (**D**) (white arrow), reflecting the malignant nature of the lesion. By histopathology, the lesion proved to be non-Hodgkin lymphoma.

**Figure 4:**
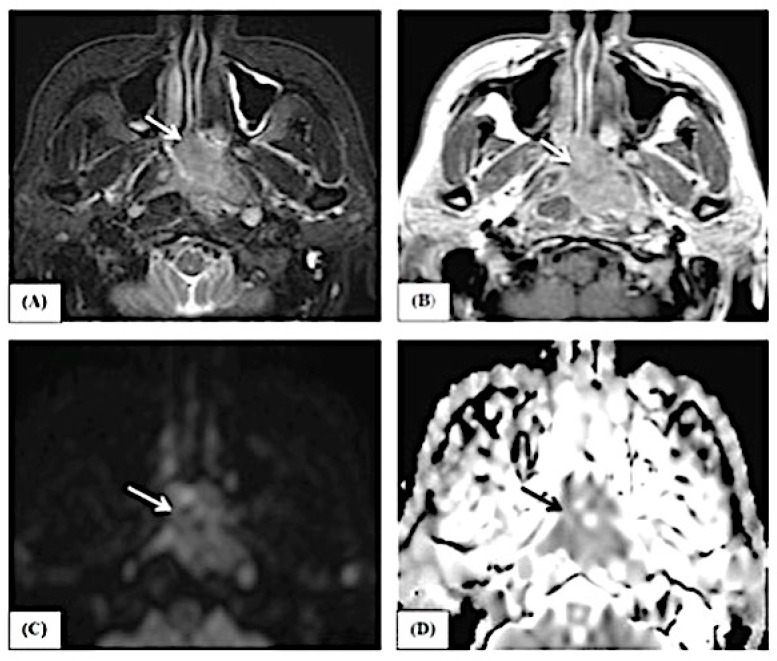
A female patient, 46 years old, complained of bleeding per nose. The axial STIR image (**A**) shows a hypointense signal of the lesion (white arrow) with nodular infiltrative margins. The axial T1 post-contrast image (**B**) shows heterogeneous enhancement (white arrow). The lesion shows restricted diffusion in the form of a hyperintense signal in B1000 (**C**) (white arrow) and a low ADC value (0.8) in the ADC map (**D**) (black arrow), reflecting the malignant nature of the lesion. By histopathology, the lesion proved to be nasopharyngeal carcinoma.

**Figure 5:**
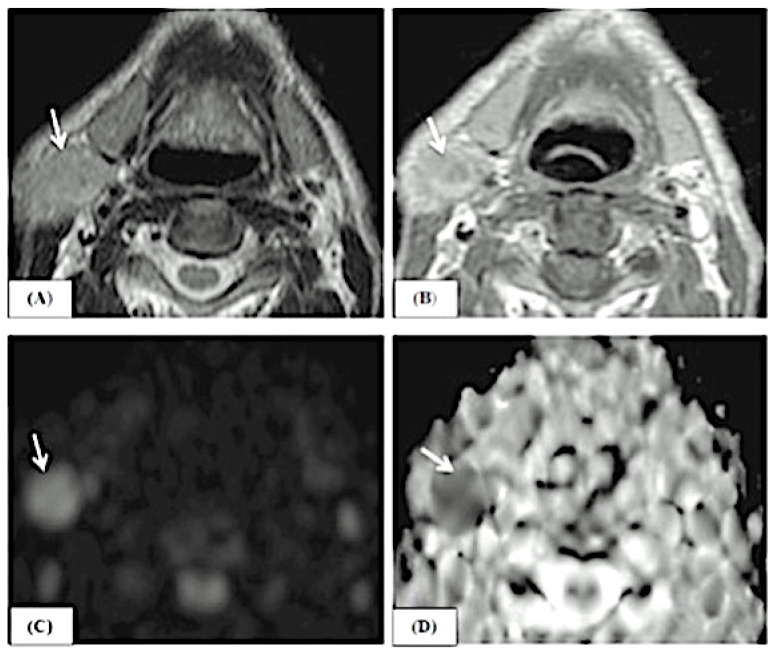
A male patient, 70 years old, complained of right-sided neck swelling. The axial T2 image (**A**) shows a hypointense signal of the right parotid lesion (white arrow) with smooth, regular margins. The axial T1 post-contrast image (**B**) shows mild enhancement (white arrow). The lesion is hyperintense in B1000 (**C**) (white arrow) and shows an ADC value of 0.5 in the ADC map (**D**) (white arrow). It was proven to be a Warthin tumor histopathologically.

This study includes examples of lesions that are depicted in conventional MRI features and ADC map pictures, as demonstrated in Figure 3, Figure 4 and Figure 5.

## 4. Discussion

The gold standard of head and neck neoformations is biopsy and histopathological diagnosis.

CT and MRI are routinely utilized in clinical practice to assess neck pathologies, with particular attention given to distinguishing between benign and malignant origins.

Establishing the benign nature of a lesion through imaging holds promise for minimizing the morbidity associated with unnecessary biopsies. Nevertheless, there often exists an overlap in imaging characteristics between benign and malignant lesions, leading to diagnostic challenges.

In such instances, measures such as the ADC, obtained from sophisticated imaging techniques like DWI, can offer supplementary, valuable information for distinguishing between different diseases. Multiple investigations have shown that non-cancerous neck conditions generally have a greater average apparent diffusion coefficient (ADC) than malignant conditions [13].

Furthermore, ADC values have been observed to differ among malignant pathologies such as SCC and lymphoma, likely attributable to variations in cellularity among different types of malignant neoplasms [14]. Our study findings demonstrate that the ADC value is a dependable indicator for distinguishing between benign, inflammatory, and malignant neck lesions.

We have expressed a wide variety of neck pathologies, most of which have been differentiated as benign or malignant based on the ADC value.

The study found that the average ADC values for malignant tumors, benign lesions, and inflammatory lesions were 1.58 ± 0.57, 0.86 ± 0.28, and 1.19 ± 0.75, respectively.

Statistical analysis revealed a significant disparity among the three cohorts (*p*, 0.001). More precisely, the average ADC values of malignant lesions were significantly lower (*p* < 0.001) compared to benign lesions. Similarly, the average ADC values of inflammatory lesions were significantly lower (*p* < 0.001) than those of benign lesions.

The investigation yielded the following statistical data: 34 true-positive findings, 1 false-positive finding, 17 true-negative findings, and 1 false-negative finding.

In our current investigation, we acquired a false-negative result in the ADC measurement for a case of thyroid cancer. This may be due to small areas of tissue death within the tumor, as proven by the histological investigation results that were not detectable on the MRI.

According to Wang et al. [15], the high apparent diffusion coefficient (ADC) values found in squamous cell carcinoma (SCC) may be attributed to the presence of tiny areas of necrosis in tumors that are not visible on magnetic resonance (MR) imaging but are confirmed through pathological inspection.

The false-positive ADC result was obtained in a case of lymphoid hyperplasia. This observation may be linked to the existence of nodal reactive changes, which are characterized by the presence of numerous germinal centers and fibrotic stroma. These changes act as microstructural obstacles.

According to ElSaid NA et al. [16], nodal reactive alterations can provide a misleading decrease in the ADC value, leading to an overestimation of the metastatic burden.

In addition, Choi KD et al. [17] proposed that limited diffusion in recent hemorrhage or hematoma could also lead to additional incorrect positive interpretations.

Consequently, it is advisable to refrain from performing DW imaging immediately after a biopsy.

This study employed a high b-value (b = 1000 s/mm^2^) to minimize the impact of capillary perfusion and water diffusion in the extracellular extravascular region.

Using larger b-values is anticipated to improve the specificity of the contrast on DWI by reducing the signal from mobile protons in the vessels. Moreover, a higher b-value magnified the disparity in the relative contrast ratios between the malignant and benign lesions.

This approach replicates the b-values utilized in investigations conducted by ElSaid et al. Studies conducted by A. Perronea et al. and K. Holzapfel et al. [16,18,19] indicated that Ali TFT [20] determined the optimal ADC threshold value for differentiating between benign and malignant nodes to be 1.15 × 10^−3^ mm^2^/s. The sensitivity, specificity, positive predictive value (PPV), negative predictive value (NPV), Kappa test, and *p*-value were reported to be 96%, 88.9%, 96%, 88.9%, 0.84, and <0.0001, respectively. Bondt et al. [21] discovered that the most effective ADC threshold value for identifying malignant cervical lymph nodes was 1.0 × 10^−3^ mm^2^/s, with a sensitivity of 92.3% and a specificity of 83.9%. The ADC cutoff values closely align with our results, which measure 1.1 × 10^−3^ mm^2^/s. 

The study conducted by ElSaid NA et al. [16] found that the average ADC value of malignant lymph nodes was 0.774 ± 0.11 × 10^−3^ mm^2^/s, while the average ADC value of benign lymph nodes was 1.019 ± 0.20 × 10^−3^ mm^2^/s. The study also identified a threshold ADC value of 1.005 × 10^−3^ mm^2^/s for distinguishing between malignant and benign nodes, with a sensitivity of 100% and a specificity of 62.5%. 

Holzapfel et al. [19] found that, by utilizing a threshold of 1.02 × 10^−3^ mm^2^/s, they were able to accurately characterize metastatic lymph nodes with a 94% accuracy rate. Their recorded average ADC values for normal and metastatic lymph nodes were 1.24 ± 0.16 × 10^−3^ mm^2^/s and 0.78 ± 0.09 × 10^−3^ mm^2^/s, respectively.

Our study has resulted in an ADC cutoff value that is lower than that previously reported in other studies. Srinivasan et al. [13] concluded from their study, conducted on 33 patients, that the cutoff ADC value was 1.3 × 10^−3^ mm^2^/s, with benign lesions having a higher ADC value and malignant lesions having a lower one. This difference is probably due to the fact that they have used a higher-field strength 3T MRI. Additionally, our study had a smaller sample size and included a diverse range of diseases, including different types of infections, which may explain the higher ADC cutoff value observed in other studies.

Wang et al. [15] found that using an ADC value of 1.22 × 10^−3^ mm^2^/s or lower resulted in the greatest accuracy of 86% for predicting malignancy.

This cutoff value was the nearest to our results (a cutoff value 1.11 × 10^−3^ mm^2^/s with an overall accuracy of 86.2%). Their study enrolled a larger number of cases (97 patients) but also used a 1.5T MRI unit. In their investigation, lymphomas exhibited notably lower ADC values compared to carcinomas, with the ADCs of carcinomas themselves being significantly lower than those of benign solid tumors. These findings align with prior research by Maeda et al. [20], which similarly reported substantially reduced ADC values in lymphomas relative to squamous cell carcinomas, in alignment with our observations (a cutoff value of 0.7, with lymphomas having lower values and squamous cell carcinomas having higher values, with an overall accuracy of 94.0%).

As far as we know, there is just one previous study by Ai S et al. [21] that has reported on the use of DWI in diagnosing tongue tumors. Their findings showed that malignant tumors had significantly lower ADC values (using b-values of 500 and 1000 s mm^2^) compared to benign lesions during 1.5-T imaging. Additionally, the ADC B5500 value of 1.43 × 10^−3^ mm^2^/s was identified as a predictor of malignancy.

Their apparent diffusion coefficient (ADC) threshold surpassed ours. This gap can be explained by several reasons, including the use of different b-values, field strengths, and differences in the histological nature of the lesions being studied. 

Regarding benign lesions, Ai et al. [21] specifically focused on cystic lesions and did not include any inflammatory lesions. In contrast, our study examined solid lesions, including inflammatory lesions, but did not include any cystic lesions. As a result, we observed a lower average ADC value.

A study conducted by S. Li et al. [22] examined tongue lesions and used receiver operating characteristic analysis to determine that an ADC threshold of 1.31 × 10^−3^ mm^2^/s provided the best ability to distinguish between benign solid lesions and malignant tumors of the tongue. This threshold achieved a sensitivity of 92.6% and a specificity of 97.3%. Furthermore, there were statistically significant differences in the ADC values observed across various forms of malignant tongue cancers (*p* < 0.001), where non-Hodgkin lymphomas displayed much lower ADC values [0.73 ± 0.08 × 10^−3^ mm^2^/s] in comparison to squamous cell carcinomas [1.12 ± 0.11 × 10^−3^ mm^2^/s] (*p* < 0.001). These results are consistent with the findings of our own investigation.

In a study conducted by Habermann et al. [23], the researchers examined parotid tumors and found that pleomorphic adenomas had average ADC values of 2.14 ± 3 × 10^−3^ mm^2^/s, Warthin tumors had average ADC values of 0.85 ± 3 × 10^−3^ mm^2^/s, and mucoepidermoid carcinomas had average ADC values of 1.04 ± 3 × 10^−3^ mm^2^/s. The observed values showed statistically significant variations when compared to other evaluated cancers (*p* < 0.001) and within each tumor type (*p* < 0.001). In contrast, there was no statistically significant variation (*p* < 0.18 to 1.00) in ADC levels across different primary malignant tumors in the parotid gland. Our results align with these findings, as we saw a Warthin tumor with an apparent diffusion coefficient (ADC) value of 0.5 and a parotid carcinoma with an ADC value of 0.7.

A study conducted by Sumi et al. [24] found that metastatic lymph nodes had significantly higher apparent diffusion coefficient (ADC) values compared to benign lymphadenopathy, while nodal lymphomas had even lower ADC levels. Metastatic nodes from highly or moderately differentiated tumors showed significantly higher ADC values compared to those from poorly differentiated malignancies. These data indicate that DWI has the ability to differentiate metastatic nodes. Abdel Razek et al. made an intriguing observation in their study [25]. They found that threshold ADC levels were useful in distinguishing between benign and metastatic nodes. However, the mean ADC values of both metastatic and lymphomatous nodes were considerably lower than those of benign nodes. These results contradict the findings of Sumi et al., who observed that metastatic nodes had greater apparent diffusion coefficient (ADC) values compared to benign nodes. The exact cause of this difference is still unknown, emphasizing the need for additional research using larger sample sizes to clarify this issue.

However, it is worth noting that the lower ADC values of nodal lymphomas compared to metastatic nodes, as demonstrated in studies by Sumi et al. and Abdel Razek et al., align more closely with our own results, wherein a cutoff value of 0.7 was observed between nodal lymphomas and metastatic squamous cell carcinoma (SCC) lymph nodes.

However, our work was subject to certain constraints. Initially, we incorporated a somewhat limited quantity of lesions. Furthermore, the consistency of the ADC results across different MR pulse sequences is still a subject of debate [26,27,28]. Therefore, the threshold level for the ADC value obtained from our findings may not be applicable to different organizations. Furthermore, the choice of ideal locations for data sampling and the identification of regions of interest (ROIs) might impact the constant collection of dependable ADC values.

## 5. Conclusions

- The qualitative evaluation of diffusion-weighted imaging (DWI) and the results obtained from the apparent diffusion coefficient (ADC) map can effectively distinguish between benign and malignant neck tumors by using a threshold value of 1.1 mm^2^/s.
- The qualitative assessment of DWI and the values of the ADC map can differentiate between squamous cell carcinoma and non-Hodgkin lymphoma with a cutoff value of 0.7 mm^2^/s.
- The quantitative assessment of the ADC value is more important than the qualitative assessment of the DWI in the characterization of the lesion.
- Adding a DWI sequence to conventional MRI in examining clinically detected neck lesions adds little to the examination time but adds much to the diagnostic performance; thus, we recommend using DWI MRI whenever there are unsolved or confusing cases by conventional imaging techniques.

## Figures and Tables

**Table 1:** Distribution of the studied cases according to complaints (*n* = 53).

Complaint	No.	%
Neck swelling	16	30.2
Lingual mass	9	17.0
Hoarseness of voice	9	17.0
Retromolar ulcer	4	7.5
Floor of mouth mass	2	3.8
Headache	2	3.8
Parotid mass	2	3.8
Bleeding per nose	1	1.9
Multiple neck swellings	1	1.9
Nasal obstruction	1	1.9
Cheek swelling	1	1.9
Upper lip mass	1	1.9
Immobile tongue	1	1.9
Mandibular mass	1	1.9
Thyroid mass	1	1.9
Submandibular mass	1	1.9

**Table 2:** Distribution of the studied cases according to the final pathology (*n* = 53).

Complaint	No.	%
Squamous cell carcinoma	19	35.8
NHL	9	17.0
Schwannoma	5	9.4
Inflammatory tongue ulcer	4	7.5
Thyroid cancer	3	5.7
Venolymphatic malformation	2	3.8
Glomus tumor	2	3.8
Lymphoid hyperplasia	2	3.8
Neurofibromas	1	1.9
Whartin	1	1.9
Inflammatory lymph node	1	1.9
Metastatic Nodal SCC	2	3.8
Parotid cancer	1	1.9
Acinic cell carcinoma of the left sublingual gland	1	1.9

NHL: non-Hodgkin’s lymphoma; SCC: squamous cell carcinoma.

**Table 3:** Comparison between the two studied groups according to the ADC value.

ADC Value (Average)	Total (*n* = 53)	Diagnosis		U	*p*
		Benign (*n* = 18)	Malignant (*n* = 35)		
Min.–Max.	0.40–2.53	0.57–2.53	0.40–2.0	86.0 *	0.001 *
Mean ± SD.	1.02 ± 0.45	1.43 ± 0.57	0.86 ± 0.28		
Median	0.93	1.43	0.87		

U: Mann–Whitney test; *p*: *p*-value for comparison between the two categories; *: statistically significant at *p* ≤ 0.05.

**Table 4:** Agreement (sensitivity and specificity) for the average ADC value to predict malignant cases.

	AUC	*P*	95% CI		Cutoff	Sensitivity	Specificity	PPV	NPV	Accuracy
			LL	UL						
Average ADC value	0.811 *	0.001 *	0.628	0.994	≤1.1	97.14	77.78	91.9	90.9	86.2

AUC: area under the curve; *p*-value: probability value; CI: confidence interval; *: statistically significant at *p* ≤ 0.05.

**Table 5:** Agreement (sensitivity and specificity) for the average ADC value to predict NHL cases (vs. Squamous cell carcinoma).

	AUC	*P*	95% CI		Cutoff	Sensitivity	Specificity	PPV	NPV	Accuracy
			LL	UL						
Average ADC value	0.965 *	<0.001 *	0.898	1.032	≤0.7	100.0	89.47	81.8	100.0	92.9

AUC: area under the curve; *p*-value: probability value; CI: confidence interval; *: statistically significant at *p* ≤ 0.05.

## Data Availability

Data is contained within the article.
